# Special Issue: Synthesis and Characterization of Graphene-Based Hybrid Nanostructures

**DOI:** 10.3390/ma14247770

**Published:** 2021-12-16

**Authors:** Zoltán Osváth

**Affiliations:** Centre for Energy Research, Institute of Technical Physics and Materials Science, P.O. Box 49, 1525 Budapest, Hungary; osvath.zoltan@ek-cer.hu

Graphene has numerous outstanding physical properties such as excellent electron mobility, extremely high thermal conductivity, high flexibility, remarkable mechanical strength, and high transparency. Combining graphene with other nanomaterials can further tune the properties of graphene, and also can enhance the characteristics of the hybrid materials produced by the addition of this monoatomic-thick carbon layer. The range of possible applications of such hybrid materials is quite vast, including but not limited to energy storage, sensing, biomedicine, photocatalysis, or optoelectronics. This Special Issue presents several of the latest advances in the synthesis and characterization of graphene-based hybrid materials and nanostructures, with relevance for the development of sodium-ion batteries [1], NO_2_ sensors [2], periodic surface structures for cell growth [3], or the long-term protection of plasmonic silver nanoparticles (NPs) [4]. The fabrication approaches, current challenges and prospects for graphene-based silicone composites are also reviewed [5].

In recent years, several intercalation materials have been investigated as potential electrodes for sodium-ion batteries (SIB). In the paper by Prosini et al. [1], the authors tested a tin-decorated reduced graphene oxide (Sn-RGO) as anode material for SIB. The Sn-RGO was coupled with a cathode material of formula NaLi_0.2_Ni_0.25_Mn_0.75_O_δ_ to form a sodium-ion cell. The so-obtained SIB was cycled at various rates and its performance was evaluated by monitoring the capacity as a function of the discharge rate and cycle number. It was confirmed that the battery was able to reversibly cycle sodium ions. The cell’s power response was good, and the cell cycled for over 100 cycles with capacities ranging from 120 up to 160 mA·h·g^−1^.

Graphene-based materials have been widely studied as sensitive layers of resistive gas sensors, as the electrical conductivity of graphene is strongly sensitive to surface adsorbates. Significant efforts were conducted to improve the response and recovery time of these sensors by controlling the functionalization of graphene. Sysoev et al. [2] reported the fluorination of few-layer graphene particles and the investigation of their sensor properties when exposed to a low concentration of nitrogen dioxide. They prepared fluorinated graphite (FG)-based films with a thickness of ~200 nm on different substrates and exposed them to 100 ppm NO_2_ at room and elevated temperatures. It was shown that the FG particle fraction affects the relative response via the efficiency of charge transfer between the NO_2_ molecules and FG layers. The authors demonstrated that highly fluorinated graphite films provided a greater increase in the sensor response as compared to less fluorinated films.

Fajstavr et al. [3] reported on the preparation of laser-induced periodic surface structures (LIPSS) on polystyrene (PS) doped with graphene nanoplatelets. Parallel ripples were attained using a laser fluence of 8 mJ·cm^−2^, while higher fluences yielded web-like surface structures. The sheet resistance and the wettability of both pristine and graphene-doped PS were significantly affected by the applied laser treatment. The construction of different LIPSS structures is very important, for example, in studies involving cell-substrate interaction, cell guidance, or tailoring protein adsorption on biomimetic surfaces.

Sensors based on the localized surface plasmon resonance (LSPR) of silver or gold nanoparticles are sensitive analytical tools which can be applied in chemical and biosensing. However, silver has poor stability under ambient conditions, forming Ag_2_S on its surface. Preserving the high LSPR intensity of silver nanoparticles is of key importance in potential applications. Our group [4] reported a method for the synthesis of graphene-covered nanoparticles and showed that Ag NPs which are completely sandwiched between graphene layers were protected from spontaneous sulphurization for at least 14 months.

In a review by Barshutina et al. [5], research from the last decade related to silicone rubber (or polydimethylsiloxane, PDMS) composites based on carbon nanotube/graphene (CNT/G) hybrid fillers is summarized. Adding CNT/G fillers to PDMS can result in highly conductive and stretchable materials with multiple potential applications in wearable electronics, medicine, or environment protection. The authors addressed key aspects such as the nanoscale architecture of CNT/G hybrid fillers, preparation methods for CNT/G/PDMS composites, and the synergistic effect of CNT/G hybrid fillers on mechanical, electrical, thermal, and other properties of silicone composites. In the last section of their paper, the authors also pointed out the major challenges that hindered the widespread use of these composite materials. These challenges include the development of effective, scalable, environmentally friendly, and safe technologies for the fabrication of seamless and highly ordered CNT/G hybrid fillers, as well as further increasing the electrical conductivity of CNT/G/PDMS composites.
